# NOX4 Mediates BMP4-Induced Upregulation of TRPC1 and 6 Protein Expressions in Distal Pulmonary Arterial Smooth Muscle Cells

**DOI:** 10.1371/journal.pone.0107135

**Published:** 2014-09-09

**Authors:** Qian Jiang, Xin Fu, Lichun Tian, Yuqin Chen, Kai Yang, Xiuqing Chen, Jie Zhang, Wenju Lu, Jian Wang

**Affiliations:** 1 State Key Laboratory of Respiratory Diseases, Guangzhou Institute of Respiratory Disease, The 1st Affiliated Hospital of Guangzhou Medical University, Guangzhou, Guangdong, China; 2 Division of Pulmonary and Critical Care Medicine, Johns Hopkins University, Baltimore, Maryland, United States of America; 3 Department of Pulmonary, Inner Mongolia People’s Hospital, Huhhot, Inner Mongolia, China; Albany Medical College, United States of America

## Abstract

**Rationale:**

Our previous studies demonstrated that bone morphogenetic protein 4 (BMP4) mediated, elevated expression of canonical transient receptor potential (TRPC) largely accounts for the enhanced proliferation in pulmonary arterial smooth muscle cells (PASMCs). In the present study, we sought to determine the signaling pathway through which BMP4 up-regulates TRPC expression.

**Methods:**

We employed recombinant human BMP4 (rhBMP4) to determine the effects of BMP4 on NADPH oxidase 4 (NOX4) and reactive oxygen species (ROS) production in rat distal PASMCs. We also designed small interfering RNA targeting NOX4 (siNOX4) and detected whether NOX4 knockdown affects rhBMP4-induced ROS, TRPC1 and 6 expression, cell proliferation and intracellular Ca^2+^ determination in PASMCs.

**Results:**

In rhBMP4 treated rat distal PASMCs, NOX4 expression was (226.73±11.13) %, and the mean ROS level was (123.65±1.62) % of that in untreated control cell. siNOX4 transfection significantly reduced rhBMP4-induced elevation of the mean ROS level in PASMCs. Moreover, siNOX4 transfection markedly reduced rhBMP4-induced elevation of TRPC1 and 6 proteins, basal [Ca^2+^]_i_ and SOCE. Furthermore, compared with control group (0.21±0.001), the proliferation of rhBMP4 treated cells was significantly enhanced (0.41±0.001) (*P*<0.01). However, such increase was attenuated by knockdown of NOX4. Moreover, external ROS (H_2_O_2_ 100 µM, 24 h) rescued the effects of NOX4 knockdown, which included the declining of TRPC1 and 6 expression, basal intracellular calcium concentration ([Ca^2+^]_i_) and store-operated calcium entry (SOCE), suggesting that NOX4 plays as an important mediator in BMP4-induced proliferation and intracellular calcium homeostasis.

**Conclusion:**

These results suggest that BMP4 may increase ROS level, enhance TRPC1 and 6 expression and proliferation by up-regulating NOX4 expression in PASMCs.

## Introduction

Pulmonary hypertension (PH) is characterized by increased mean pulmonary arterial pressure (mPAP, at resting condition) >25 mmHg. PH is a progressively developing disease and eventually leads to right heart failure and death [1]. Many studies have confirmed that vascular stenosis is a main characteristic of PH and is caused by excessive distal small pulmonary arterial remodeling, and further develops into the increase in pulmonary vascular resistance, leads to increased right ventricular overload and eventually causes right ventricular heart failure, even death [1]. Further study demonstrated that ROS (reactive oxygen species) plays an important role in pulmonary vascular proliferation and remodeling in chronic hypoxic pulmonary hypertension (CHPH) [2]. ROS is generated by electrons passing through biological membranes induced by NADPH (nicotinamide-adenine dinucleotide phosphate) oxidase NOX4 [3]. Numerous studies indicated that BMP4 (bone morphogenetic protein 4), a multifunctional ligand which belongs to the transforming growth factor β superfamily, could promote the proliferation, and inhibit the apoptosis of PASMCs [4], [5], [6], [7]. So, BMP4 is thought as a crucial contributor to CHPH development. Others and our previous studies have shown that the hypoxia-elevated proliferation is largely due to enhanced intracellular Ca^2+^ concentration ([Ca^2+^]_i_), moreover, the enhanced basal [Ca^2+^]_i_ is mediated by hypoxia triggered store-operated calcium entry (SOCE) via store-operated calcium channel (SOCCs) [8], [9]. SOCCs is primarily composed by transient receptor potential channel (TRPC) [8], [10]. Among the seven members of TRPC, TRPC1, TRPC4 and TRPC6 are most abundantly expressed in distal pulmonary artery and PASMCs, whereas, TRPC1, TRPC6 expressions are selectively upregulated by hypoxia [8], [11], [12]. Moreover, it was confirmed that TRPC1 and TRPC6 are essential for the CHPH pathogenesis [13], [14]. In PASMCs, BMP4 up-regulates TRPC1 and 6 expressions in rat pulmonary artery and PASMCs to increase [Ca^2+^]_i_ and SOCE, further leads to increased proliferation, which leads to pulmonary small artery spasm contraction and remodeling, and eventually causes elevated pulmonary resistance and PH [6], [15]. However, it still remains largely unclear how BMP4 induces TRPCs expression. Recent studies have confirmed that TGF-β-induced NOX4 expression and ROS generation were significantly associated with the proliferation of PASMCs [16]. Similarly, we sought to wander: 1) whether BMP4, also functions as a multiple faces’ factor, could influence ROS generation and NOX4 expression? 2) whether such induction controls the downstream TRPC expression and the intracellular calcium homeostasis? 3) whether these mechanisms fit into and explain the mechanisms through which BMP4-induced PASMCs proliferation and pulmonary vascular remodeling? This study aims to clarify the mechanism underlying BMP4 regulating calcium homeostasis and pulmonary vascular remodeling in PASMCs, to provide a theoretical basis for the subsequent development of drugs for the treatment.

## Materials and Methods

### Reagents and Instruments

Sprague Dawley (SD) rats (weight 250 g–300 g) were purchased from Guangdong Experimental Animal Center and housed under standard specific pathogen free (SPF) conditions; All procedures were in accordance with National Institutes of Health guidelines for use of live animals and approved by the Institutional Animal Care and Use Committee (IACUC) of Guangzhou Medical University, Guangzhou, China [License No.: SCXK (Guangdong) 2008–0002]. All surgery was performed under anesthesia with sodium pentobarbital (65 mg/kg i.p.), and all efforts were made to minimize animal suffering.

Fetal bovine serum (FBS), DMEM culture medium was purchased from Hangzhou GINO Co. Ltd, DCFH-DA from U.S. Molecular Probes Company. Fura-2 dye from U.S. Invitrogen Company, BMP4 recombinant protein was purchased from U.S. R&D Company, NOX4 monoclonal anti-rabbit antibody from UK Abcam company, TRPC1 and 6 polyclonal anti-rabbit antibody from Israeli Alomone Labs company, α-actin monoclonal anti-rats antibody from American Santa cruz company, HRP-conjugated goat-anti-rabbit secondary antibody and goat-anti-mouse secondary antibody were purchased from American KPL Company. Acrylamide, methylene bis-acrylamide, ammonium persulfate, Tris base, glycine, sodium dodecyl sulfate (SDS), polyvinylidene fluoride (PVDF) membranes, enhanced chemiluminescent (ECL) chemiluminescence solution and protein electrophoresis transfer systems were purchased from U.S. Bio-rad Company. NOX4 siRNA oligo was synthesized by Shanghai GenePharma Co. Ltd. GenSilencersiRNA transfection kit was purchased from American Genlantis Company. MTT reagent and DMSO were purchased from Sigma, U.S., RIPA cell lysates and 30% H_2_O_2_ from China Biyuntian Biotechnology Institute. Other reagents were of analytical grade products. Flow cytometry Beckman Corporation and full wavelength scanners were purchased from U.S. Thermo Electron Corporation.

### PASMCs Culture and Identification

The PASMC culture and identification methods were the common methods used in our study group [11], [12].

### Recombinant BMP4 Protein Treatment

The primary PASMCs were cultured with 10% FBS DMEM medium until 70–80% confluence. The growth medium was then replaced by 0.5% FBS low glucose DMEM medium to starve for 24 hours. After appropriate starvation, cells were randomly divided into two groups and treated with either vehicle control for the control group or with 50 ng/ml BMP4 recombinant protein for 24 hours.

### Detection of Oxygen Free Radicals in PASMCs

After proper culture, the cells were digested by 0.25% trypsin (without EDTA) and collected, then centrifuged at 1000 rpm for 5 minutes and supernatant was discarded. The cells were washed with sterile PBS 1–2 times and suspended with 1 ml PBS solution with 5 µM DCFH-DA. After incubation for 30 minutes at 37°C away from light, samples were centrifuged at 1000 rpm for 5 minutes, washed with sterile PBS for 1–2 times. Cells were finally suspended in 0.5 ml PBS and performed flow cytometry assay. DCFH-DA enters the cells, which, after lipase hydrolysis, becomes DCFH. Non-luminous DCFH breaks into fluorescent substance DCF through oxygen free radicals in the cells. As a result, there is a proportional relationship between DCF fluorescence intensity and the content of oxygen free radicals in cells. DCF excitation wavelength is 488 nm, absorption wavelength 530 nm.

### Western blot

After proper culture and treatment, cells were lysed and total protein was extracted by RIPA buffer. To determine cell protein concentration, with α-actin as internal control, 40 µg sample protein from each group was conducted electrophoresis by using 12% SDS-PAGE concentrating gel at 120V for 10 min and separating gel at 150 V for 90 min, transfected by electroporation transfer film in ice, blocked with 5% milk-TBST for 1 hour, and incubated with primary antibody overnight, followed by secondary antibody incubation for 1 hour. Bound antibodies were finally detected using an enhanced chemiluminescence system.

### Small interference RNA Transfection

Two pairs of siRNA were synthesized as follows: NOX4-rat-205 (sequence: 5′ AGGAUUGUGUUUGAGCAGATT 3′, 5′ UCUGCUCAAACACAAUCCUTT 3′), and NOX4-rat-1048 (sequence: 5′ GACCUGGCCAGUAUAUUAUTT 3′, 5′ AUAAUAUACUGGCCAGGUCTT 3′). While designing one pair of test sequences for negative control group (sequence: 5′ UUCUUCGAACGUGUCAGGUTT 3′, 5′ ACGUGACACGUUCGGAGAATT 3′). Primarily cultured cells at 50–70% confluence were transfected with siNOX4 or nontargeting control siRNA using GenSilencer siRNA transfection reagent as carrier for 4 hours in serum-free DMEM in 5% CO_2_ at 37°C. The final concentration of each siRNA was 1000 ng/ml. 8 µl 10% FBS was then added and the cells were incubated for 72 hours before protein, flow cytometry, or MTT assay. Recombinant BMP4 Protein (50 ng/ml) and H_2_O_2_ (100 µM) [17], [18] treatment need to be included after siRNA transfection 48 h. Then after 24 h treatment with recombinant BMP4 Protein and H_2_O_2_, proceed protein and intracellular Ca^2+^ determination experiments.

### MTT cell proliferation measurement

Cells were grouped as mentioned above, digested with 0.25% trypsin and dispersed with a medium containing 0.5% FBS to a concentration of 50,000 cells/ml as a single cell suspension. 10,000 cells (200 µl single cell suspension) from each group were seeded in 96-well cell culture plate, with 5 parallel wells for each group. To avoid edge effects, an equal volume of PBS was added around the filled wells in 5% CO_2_ at 37°C (within 24 hours). After attachment, discard medium solution and add MTT solution to each well (the final concentration was 5 mg/ml), incubate again in darkness in 5% CO_2_ incubator at 37°C for 4–6 hours. Discard supernatant, add 150 µl DMSO to each well, vibrate the plate for 10 minutes until crystalline solid was totally dissolved. Scan and determine the optical density value (Optical density, OD) by Full wavelength scanner at wavelength of 490 nm. Experiments were repeated three times.

### Intracellular Ca^2+^ concentration measurement

SOCE was measured in PASMCs using Fura-2 dye and fluorescent microscopy as previously described [6], [7], [8], [19]. To obtain statistically convincing results, the fluorescence intensity was determined in at least 30 cells for each sample.

### Statistical Analysis

Gray values were analyzed using ImageJ software. Experimental data, as shown in 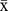
±S, were analyzed using statistical software SPSS13.0. Two samples were compared using the t test, multiple groups were compared using univariate analysis of variance (one way-ANOVA) F test, pairwise comparisons between groups using LSD method. P<0.05 was considered statistically significant.

## Results

### Recombinant BMP4 protein induced NOX4 protein expression and ROS mean level in rat distal PASMCs

**Figure 1:**
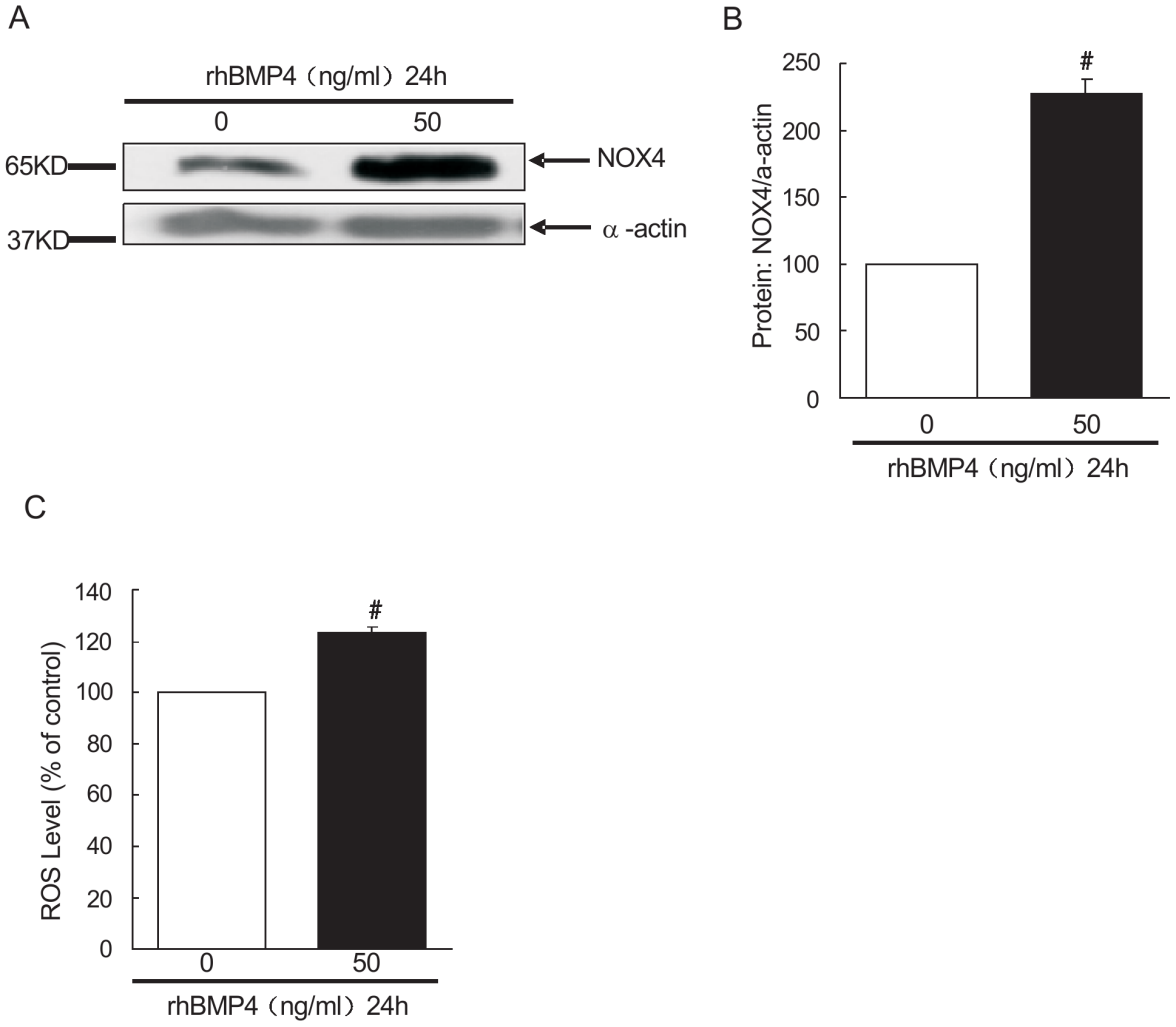
Bone morphogenetic protein 4 (BMP4) increased NOX4 NADPH oxidase 4 (NOX4) expression and ROS mean level in PASMCs. A: Western blot analysis of BMP4 protein impacting NOX4 in rat distal PASMCs, of which the upper band is NOX4, the following band is α-actin. Control group and BMP4 group indicate blank control group and recombinant BMP4 (50 ng/ml for 24 hours) group respectively. B: BMP4 effects on NOX4 protein in rat distal PASMCs (X±S, n = 3) #*P*<0.01 vs. control group, the results have significant difference. C: BMP4 induced reactive oxygen species (ROS) generation in rat distal PASMCs. Control group and BMP4 group indicate blank control group and recombinant BMP4 (50 ng/ml for 24 hours) group respectively. (X±S, n = 3) #*P*<0.01 vs. control group, the results have significant difference.

Cultured rat distal PASMCs were treated with rhBMP4 or respective vehicle control. The cells without rhBMP4 treatment serve as control group. After 24 hours, expression of NOX4 protein was assessed by western blotting. As shown in Figure 1 A and B, the NOX4 protein expressions in cells incubated with the recombinant BMP4 protein (50 ng/ml) for 24 hours were (226.73±11.13) % relative to those in control group (*P*<0.01), the difference was statistically significant. The ROS mean level in the PASMCs from rhBMP4 treated group and control group was assayed using Flow Cytometry. As shown in Figure 1C, the ROS mean level in cells incubated with the recombinant BMP4 protein (50 ng/ml) for 24 hours was (123.65±1.62) %, relative to that in control group (*P*<0.01), the difference was statistically significant.

### NOX4-knockdown abolished BMP4-induced ROS production in rat distal PASMCs

**Figure 2:**
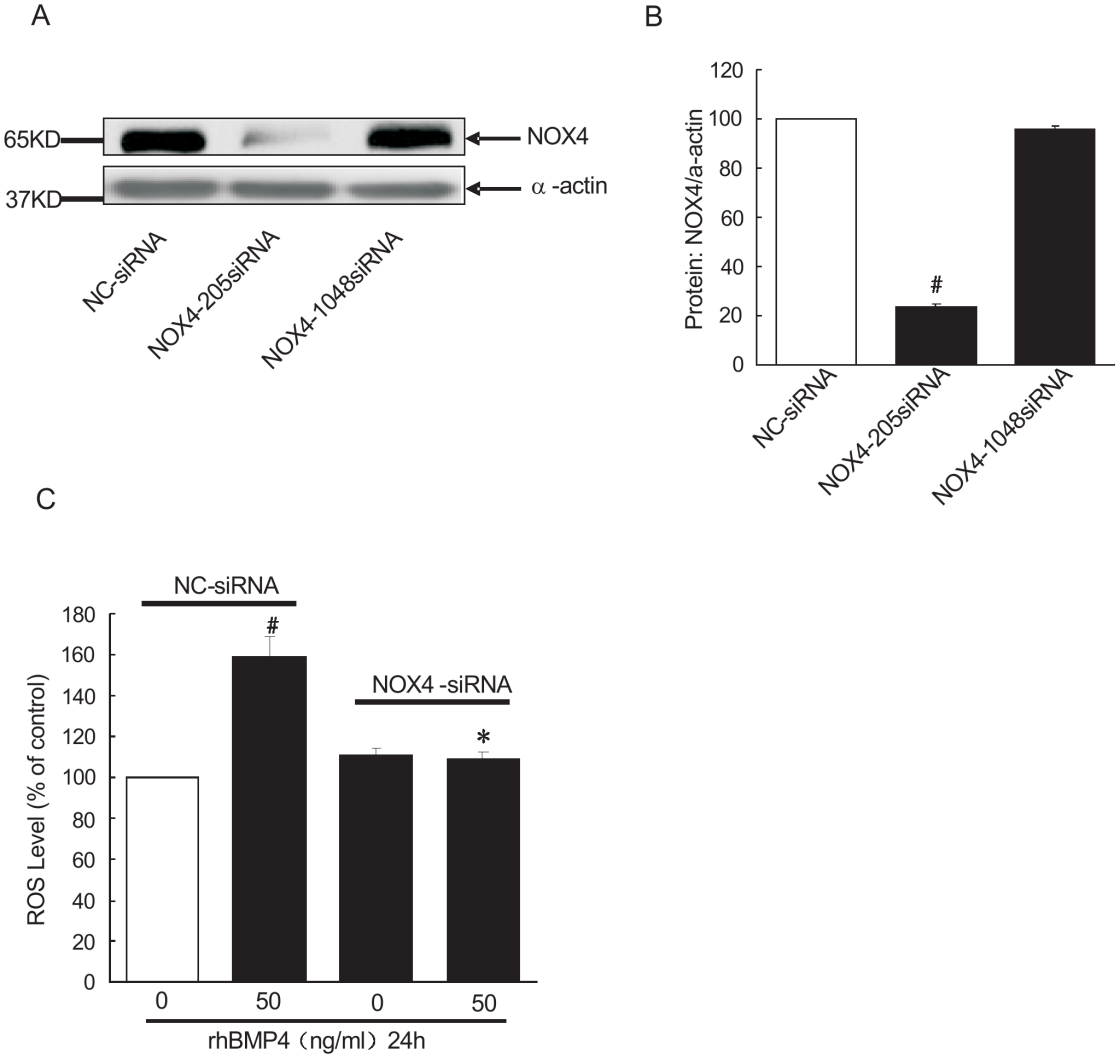
NOX4 knockdown downregulated BMP4 induced ROS generation in rat distal PASMCs. A: Western blot analysis of NOX4 siRNA affecting NOX4 protein in rat distal PASMCs, of which the upper band is NOX4, the following band is α-actin. NC group, NOX4-205 siRNA group and NOX4-1048 siRNA group indicate negative control group, NOX4 siRNA group respectively. B: NOX4 siRNA affects NOX4 protein expression in rat distal PASMCs (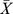
±S, n = 4). #indicates *P*<0.01 vs. negative control group, the results have significant difference. C: From left to right are negative control group, negative control+BMP4 group, NOX4 siRNA group, and NOX4 siRNA+BMP4 group respectively. (X±S, n = 3), #indicates *P*<0.01 vs. NC-siRNA group, *p<0.01 vs. NC-siRNA plus BMP4 group.

To evaluate the role of NOX4 on BMP4-induced ROS induction in PASMCs, we adopted a loss-of-function approach by knocking down the endogenous expression of NOX4 using its specific siRNA. We firstly synthesized siRNA oligo targeting NOX4 (NOX4-rat-205 siRNA, NOX4-1048 siRNA) and transfected PASMCs to verify the knockdown specificity. Non-targeting siRNA (NC-siRNA) was used as a non-targeting control. As shown in Figure 2A and B, after transfection with NOX4-rat-205 siRNA oligo, NOX4 protein expression decreased to (23.30±1.62) %, relative to that in negative control group (*P*<0.01), the difference was statistically significant. However, in NOX4-1048 siRNA transfected group, NOX4 expression was (95.53±1.51) %, relative to that in negative control group (*P*>0.05). The difference was not statistically significant. We confirmed that NOX4-rat-205 siRNA was the specific NOX4-siRNA. Next, cultured rat distal PASMCs were divided into four groups: 1) NC-siRNA treated group serves as negative control; 2) NC-siRNA transfected plus BMP4 treatment group; 3) NOX4 siRNA transfection group and 4) NOX4 siRNA transfection plus BMP4 treatment group. Afterwards, the ROS mean levels in each group were detected by flow cytometry. As shown in Figure 2C, rhBMP4-treatment after transfection with NC-siRNA, the ROS mean level in rat PASMCs was (158.88±9.58) % relative to that in negative control group (*P*<0.01), the difference was statistically significant. However, knockdown of NOX4 markedly abolished BMP4 induced ROS mean level, which was (108.23±4.07) % relative to that in negative control group. *p<0.01 compare to NC-siRNA plus BMP4 group. The ROS mean level in those transfected by NOX4-siRNA without BMP4 treatment was (110.65±3.78) % relative to that in negative control. There was no significant difference in ROS levels between negative control siRNA group and NOX4-siRNA transfection group (*P*>0.05).

### Knockdown of NOX4 attenuated BMP4-induced up-regulation of TRPC1 and 6 in rat distal PASMCs

**Figure 3:**
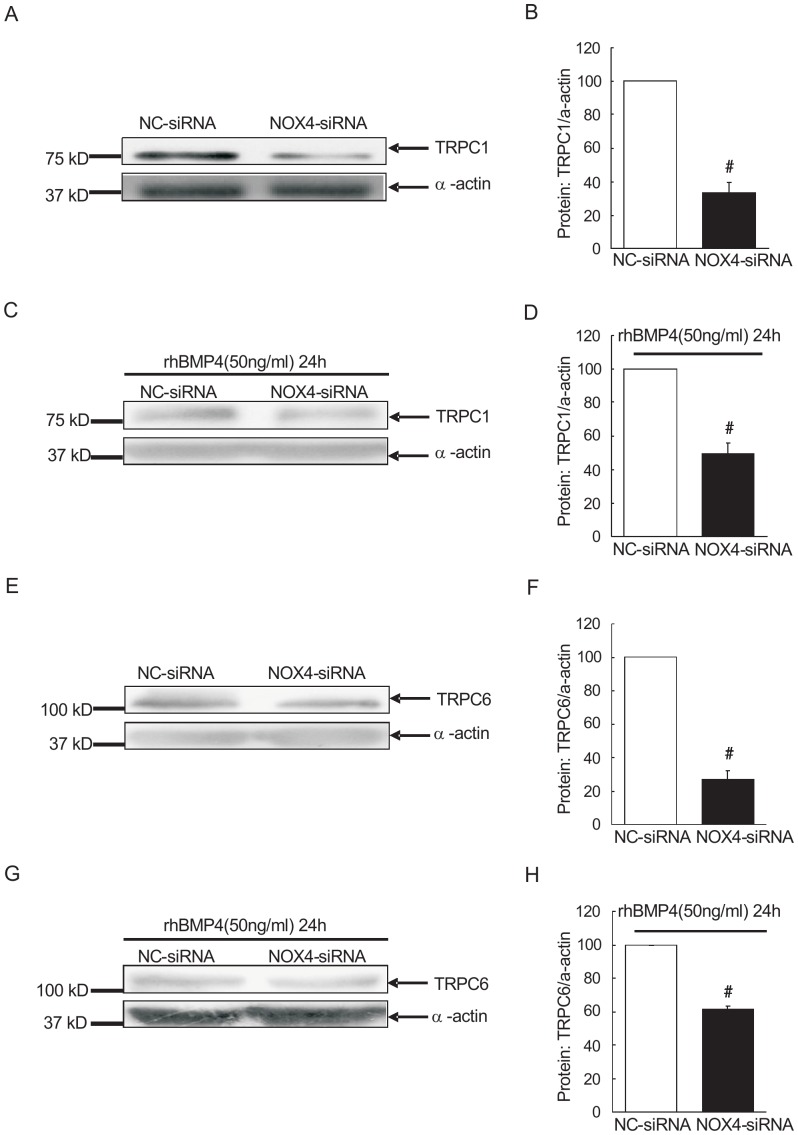
NOX4 knockdown decreased TRPC1 and TRPC6 expression under both control and BMP4 treatment circumstances. A: Western blot analysis of TRPC1 protein after knockout of NOX4 in rat distal PASMCs, of which the upper band is TRPC1, the following band is α-actin; NC and NOX4 siRNA group indicate negative control group and NOX4 knockout group respectively. B: results of knockout of NOX4 affecting TRPC1 protein in rat distal PASMCs. (X±S, n = 3), #indicates *P*<0.01 vs. Negative control group, the results have significant difference. C: Western blot analysis of BMP4 affecting TRPC1 in rat distal PASMCs after knockout of NOX4, of which the upper band is TRPC1, the following band is α-actin. NC+BMP4 group, NOX4 siRNA+BMP4 group indicate negative control group and NOX4-knockout with recombinant BMP4 treatment (for 24 hours) group, respectively. D: Results of BMP4 affecting TRPC1 expression in rat distal PASMCs after knockout of NOX4. (X±S, n = 3), #indicates *P*<0.01 vs. Negative control+BMP4 group, the results have significant difference. E: Western blot analysis of TRPC6 protein after knockout of NOX4 in rat distal PASMCs, of which the upper band is TRPC1, the following band is α-actin; NC and NOX4 siRNA group indicate negative control group and NOX4 knockout group respectively. F: results of knockout of NOX4 affecting TRPC1 protein in rat distal PASMCs. (X±S, n = 3), #indicates *P*<0.01 vs. Negative control group, the results have significant difference. G: Western blot analysis of BMP4 affecting TRPC6 in rat distal PASMCs after knockout of NOX4, of which the upper band is TRPC6, the following band is α-actin; NC+BMP4 NOX4 siRNA+BMP4 group indicate negative control group and NOX4-knockout with recombinant BMP4 treatment (for 24 hours) group, respectively. H: Results of BMP4 affecting TRPC6 expression in rat distal PASMCs after knockout of NOX4. (X±S, n = 3), #indicates *P*<0.01 vs. Negative control+BMP4 group, the results have significant difference.

Since previous studies indicated that BMP4 up-regulates the expression of TRPCs in PA and PASMCs, we want to determine whether NOX4 fits into the signaling cascade and mediates the BMP4-induced TRPCs expression. As shown in Figure 3A and B, TRPC1 expression in PASMCs of NOX4 siRNA group was (33.39±5.95) %, relative to that in negative control siRNA group (100±0%). We further treated both groups with recombinant BMP4 (50 ng/ml), then assess TRPC1 protein expression by western blotting. As shown in Figure 3 C and D, relative expression of TRPC1 in NOX4-siRNA+BMP4 group was 49.72±6.05% relative to NC-siRNA+BMP4 group. Moreover, TRPC6 showed similar pattern as TRPC1 in the expression level in all the treatment groups: NC-siRNA group, NOX4-siRNA group, NC-siRNA+BMP4 group and NOX4-siRNA+BMP4 group. As shown in Figure 3E, F, G and H, TRPC6 protein expressions in NOX4-siRNA transfected PASMCs was 27.01±5.33% relative to that in NC-siRNA control, with a statistically significant difference (*P*<0.01). TRPC6 protein expression in NOX4-siRNA group was (61.19±2.33%), also markedly lower than that in NC-siRNA group (100±0%), which had a statistically significant difference (*P*<0.01). It indicated that NOX4 mediate BMP4-induced TRPC1 and TRPC6 protein expression.

### Knockdown of NOX4 blocked BMP4-induced proliferation in rat distal PASMCs

**Figure 4:**
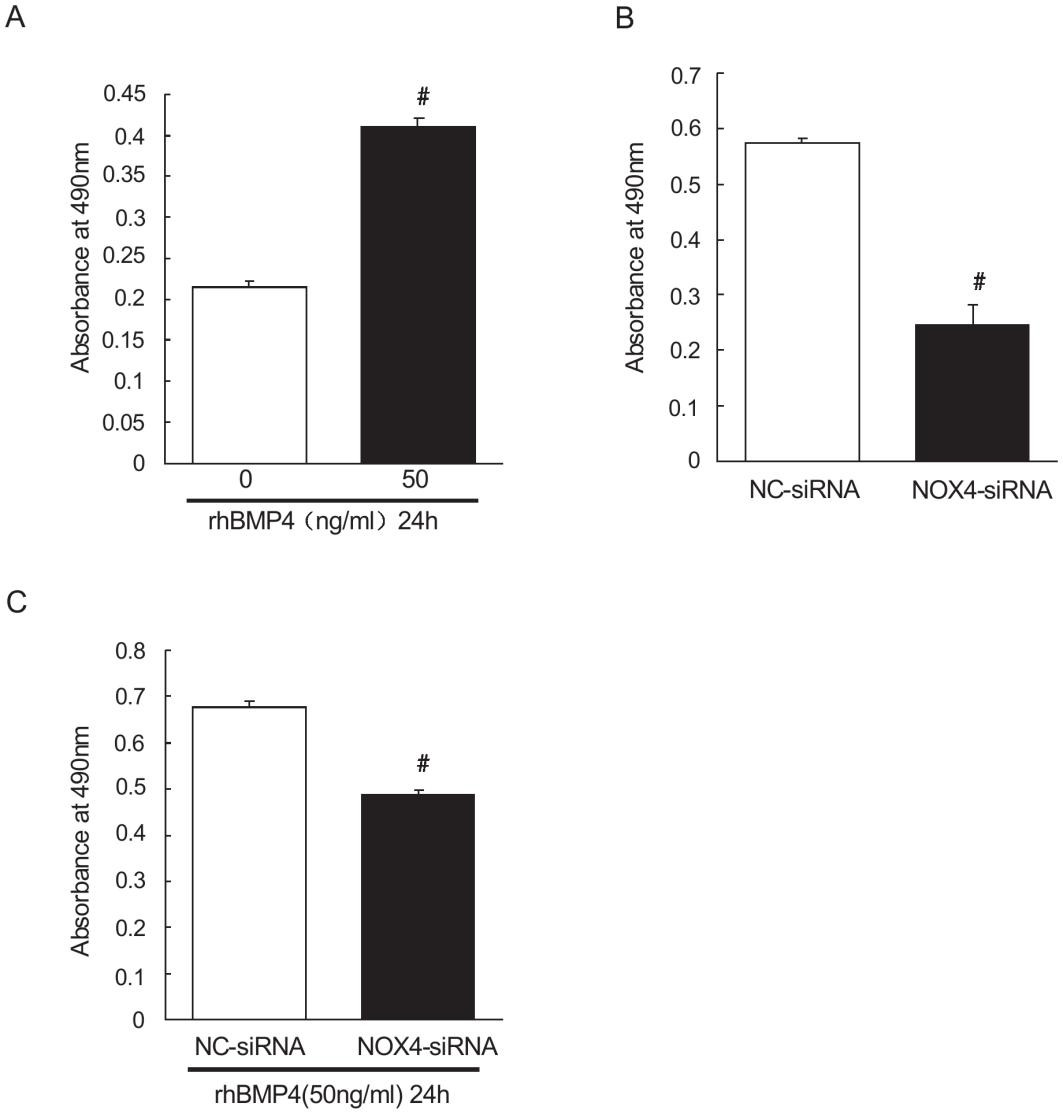
NOX4 knockdown inhibits proliferation of PASMCs under both control and BMP4 treatment circumstances A: control group was the control group absorbance at 490 nm, BMP4 group was absorbance of recombinant BMP4 protein intervention for 24 h group at 490 nm, #indicates P<0.01 vs. control group, the results are statistically significant. B: absorbance of rat distal PASMCS at 490 nm after transfection with NOX4 siRNA and knockout of NOX4, NC indicates negative control group; NOX4 siRNA indicates NOX4 gene silencing group; #indicates *P*<0.01 vs. negative control group, results have statistically significance. C: absorbance of rat distal PASMCS at 490 nm after transfection with NOX4 siRNA and knockout of NOX4, and treatment with recombinant BMP4 for 24 hours. NC+BMP4 group indicates negative control+BMP4 treatment group; NOX4 siRNA+BMP4 group was BMP4 with silent NOX4 group, #indicates *P*<0.01 vs. negative control+ BMP4 group, the results are statistically significant.

We further observed the effect of NOX4 on the proliferation of PASMCs. As shown in Figure 4A, B and C, the absorbance values at wavelength of 490 nm in control group and BMP4 group were 0.21±0.001, 0.41±0.01 respectively. Compared with that in control group, there was a statistically significant increase in BMP4 group (treatment with recombinant BMP4 for 24 hours, *P*<0.01). After transfection with negative control siRNA and NOX4-siRNA, the absorbance values at wavelength of 490 nm were 0.57±0.01 and 0.24±0.03 respectively. However, when treated with recombinant BMP4 for 24 hours after transfection, the absorbance values of PASMCs at wavelength of 490 nm were 0.67±0.01 and 0.48±0.01 respectively. It indicated that compared with control group, knockdown of NOX4 significantly inhibits PASMCs proliferation (*P*<0.01). However, BMP4 did not reverse this process. After NOX4 knockdown and recombinant BMP4 treatment, cell proliferation was still significantly inhibited, relative to that in control group (*P*<0.01).

### Knockdown of NOX4 inhibited BMP4-induced increase of basal [Ca^2+^]_i_ and SOCE in rat distal PASMCs

**Figure 5:**
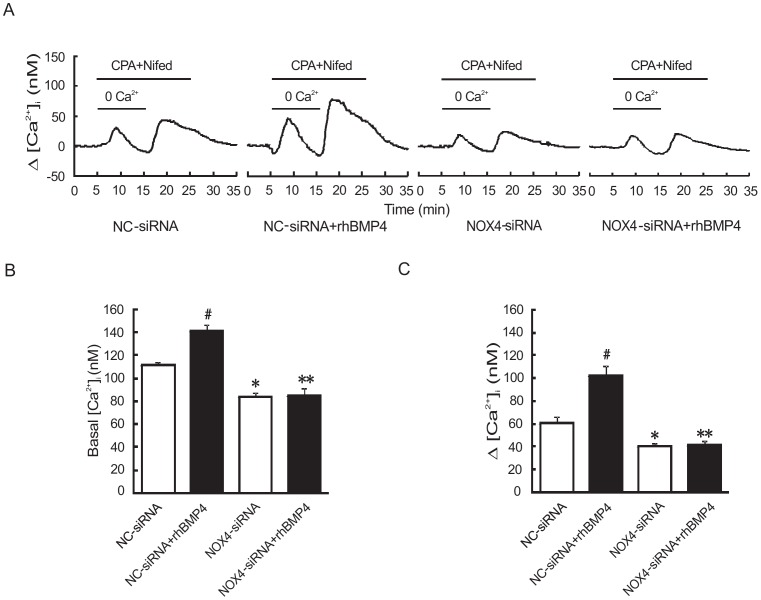
NOX4 knockdown downregulate basal [Ca^2+^]_i_ and SOCE of rat distal PASMCs in the condition with or without BMP4 treatment. A and C: changes of SOCE in PASMCs from NC-siRNA group, NC-siRNA+rhBMP4 group, NOX4-siRNA group, NOX4-siRNA+rhBMP4 group (X±S, n = 5, #indicates *P*<0.01 vs. NC-siRNA group, the results are statistically significant. *indicates *P*<0.01 vs. NC-siRNA group, the results are statistically significant. **indicates *P*<0.01 vs. NC-siRNA group and NC-siRNA+rhBMP4 group, the results are statistically significant). B: changes of basal [Ca^2+^]_i_ in four group (X±S, n = 5, #indicates *P*<0.01 vs. NC-siRNA group, the results are statistically significant. *indicates *P*<0.01 vs. NC-siRNA group, the results are statistically significant. **indicates *P*<0.01 vs. NC-siRNA group and NC-siRNA+rhBMP4 group, the results are statistically significant.).

Since NOX4-siRNA can block BMP4-induced TRPC1 and 6 protein expression, we then sought to figure out whether NOX4-siRNA inhibits TRPC1 and 6 activity in their SOCE mediation major and the intracellular Ca^2+^ regulation. As shown in Figure 5A, B and C, after transfection with NC-siRNA, pre-treatment with rhBMP4 (50 ng/ml) increased basal [Ca^2+^]_i_ (141.18±5.85 nM) and SOCE (102.39±8.48 nM) in rat distal PASMCs, compared with NC-siRNA without BMP4 treatment group (111.43±2.63 nM, 60.79±5.20 nM), and this had a statistically significant difference (*P*<0.01). However when transfection with NOX4-siRNA, basal [Ca^2+^]_i_ and SOCE were 83.91±3.94 nM and 40.70±2.02 nM respectively, which obviously decreased compared with NC-siRNA group, and the difference was statistically significant (*P*<0.01). These results were not altered after pre-treatment with rhBMP4 in NOX4-siRNA group (85.45±5.80 nM, 41.64±3.69 nM), suggesting BMP4 regulates intracellular Ca^2+^ homeostasis through altering NOX4.

### External ROS rescues the effects of NOX4 knockdown in rat distal PASMCs

**Figure 6:**
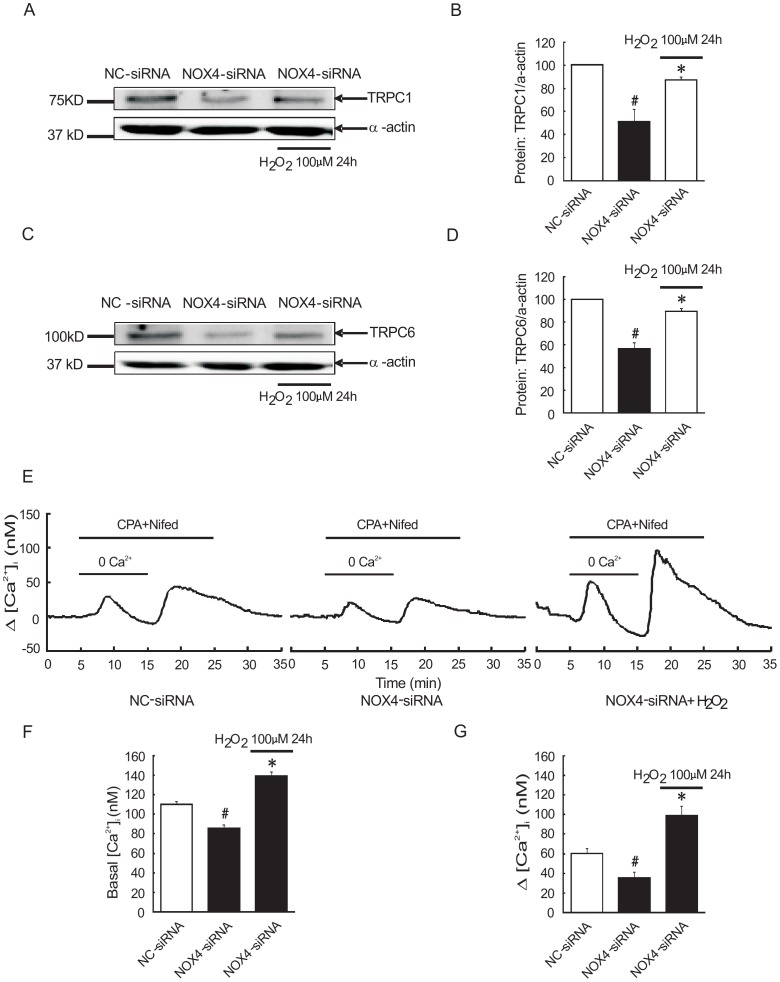
H_2_O_2_ reversed the effect of knockdown NOX4 upregulated TRPC1 and TRPC6 expression, basal [Ca^2+^]_i_ and SOCE in rat distal PASMCs. A–D: expression levels of TRPC1 and TRPC6 protein were detected by western blot (A, C: representative blots, B, D: mean intensity for TRPC1 and TRPC6) (X±S, n = 4, #indicates *P*<0.01 vs. NC-siRNA group, the results are statistically significant. *indicates *P*<0.01 vs. NOX4-siRNA group, the results are statistically significant.) F: basal [Ca^2+^]_i_ in PASMCs of three group (NC-siRNA group, NOX4-siRNA group, NOX4-siRNA+ H_2_O_2_ group) (X±S, n = 4, #indicates *P*<0.01 vs. NC-siRNA group, the results are statistically significant. *indicates *P*<0.01 vs. NC-siRNA group and NOX4-siRNA group, the results are statistically significant.) E, G: changes of SOCE in NC-siRNA group, NOX4-siRNA group and NOX4-siRNA+ H_2_O_2_ group. (X±S, n = 5, #indicates *P*<0.01 vs. NC-siRNA group, the results are statistically significant. *indicates *P*<0.01 vs. NC-siRNA group and NOX4-siRNA group, the results are statistically significant.).

From all the results above, we knew that knockdown of NOX4 abolished BMP4-induced ROS generation and TRPC expression. So, what happen when external ROS are involved? As shown in Figure 6A, B, C and D, transfection with NOX4-siRNA TRPC1 and TRPC6 protein expression in PASMCs were (56.58±5.36) % and (51.23±10.58) % respectively, relative to that in NC-siRNA group ((100±0) % and (100±0) %). But pretreatment with H_2_O_2_ (100 µM) reversed this effect, TRPC1 and TRPC6 expression in NOX4-siRNA+ H_2_O_2_ group were (89.60±2.58) % and (87.08±2.68) %, relative to NC-siRNA group. Moreover, we tested basal [Ca^2+^]_i_ and SOCE in NC-siRNA group, NOX4-siRNA group and NC-siRNA+ H_2_O_2_ group respectively. As shown in Figure 6E, F and G, basal [Ca^2+^]_i_ and SOCE in NC-siRNA group were 110.16±3.30 nM and 60.59±5.43 nM. However, the NOX4-siRNA group were 85.76±3.82 nM and 35.76±5.44 nM, and their difference was statistically significant (P<0.01). Contrast with NOX4-siRNA group, the basal [Ca^2+^]_i_ and SOCE of NC-siRNA+ H_2_O_2_ group increased into 139.89±4.40 nM and 99.16±9.58 nM, which had a statistically significant (P<0.01).

## Discussion

In this study, we found that rhBMP4 increased NOX4 protein expression and ROS mean level in rat distal PASMCs, and further investigated the role of NOX4 with a loss of function strategy using siRNA. To confirm whether NOX4 regulated intracellular Ca^2+^ homeostasis via affecting ROS generation, we applied external ROS (H_2_O_2_) to rescue the ROS level, under the condition of NOX4 knockdown. Our results showed that rhBMP4-induced ROS generation and TRPCs protein up-regulation, basal [Ca^2+^]_i_ and SOCE increase, as well as excessive cell proliferation, are dependent on BMP4-induced NOX4 expression.

During the recent years, proportional BMPRII gene mutation has been found in idiopathic pulmonary arterial hypertension (IPAH) and HPAH (familial PAH) patients in Chinese Han people [20]. Further studies have revealed that combination of BMP with BMPRI and BMPRII may cause dependent phosphorylation of the receptor of cytosolic inhibitors–Smad1, Smad5, Smad8. Phosphorylated Smads then interact with the co-Smad, Smad4, regulate the target gene transcription, including Smad binding sequence [21], [22]. Xudong Yang, etc. [4] found that BMP4 plays a key regulatory role in the proliferation of the PASMCs isolated from patients with familial pulmonary hypertension via p38MAPK and ERK1/2 signaling pathway. L. Dewachter, etc. [23] confirmed that in idiopathic pulmonary hypertension, BMP4 promotes PASMCs mitosis through Smad and p38MAPK pathway. Our previous study [6] has found that BMP4 can induce TRPC1 and 6 up-regulation by activating the p38MAPK and ERK1/2 pathway in PASMCs, promoting SOCE through activating TRPC channels and increasing basal [Ca^2+^]_i_, thus contributing to pulmonary vasoconstriction and PASMCs proliferation, eventually leading to pulmonary vascular remodeling in rats. In the present study, we further confirmed that recombinant BMP4 protein (50 ng/ml) could promote the expression of TRPC1 and 6, basal [Ca^2+^]_i_, SOCE and the proliferation of rat distal PASMCs.

Then, we sought to determine through which signaling pathway does BMP4 induce TRPC1 and 6 expressions, alter intracellular calcium homeostasis and cell proliferation. Many studies focusing on the endothelial function revealed a tight relationship among BMP4, NOX4 and ROS. Xiao Yu Tian, etc. confirmed that BMP4 leads to endothelial cell apoptosis through NADPH oxidase/ROS/p38MAPK/JNK pathway [24]. By using small interfering RNA gene silencing BMP4, the shear stress-induced release of ROS in endothelial cells was inhibited, which is mainly caused by NOX1 [25]. In addition, compared with the low-fat diet, high-fat diet could increase NOX4 protein and increase its signaling pathway upstream BMP4 expressions in C57BL/6 mouse thoracic aorta tissue [26]. These results indicated that BMP4 plays an important role in regulating NOX4 and ROS levels. We firstly tested whether BMP4 promotes NOX4 and ROS levels in rat distal PASMCs. Results showed that NOX4 and ROS were increased by BMP4 treatment and the increased ROS level could be attenuated through interfering endogenous NOX4 gene using siRNA knockdown. This indicates that in PASMCs, BMP4 increased ROS generation, at least in part, is mediated by NOX4. ROS plays an important role in occurrence and development of CHPH, mainly in the following three aspects: 1) regulates proliferation and apoptosis activity; 2) participates in pulmonary artery smooth muscle contraction; 3) regulates hypoxic pulmonary vascular remodeling. This study confirmed that NOX4, the highest expressed NADPH oxidase in PASMCs, plays a role in electron transfer in the process of hypoxia-induced increase in ROS levels. In the lung tissue specimens of patients with idiopathic pulmonary hypertension, NOX4 was significantly increased, mainly localized in the blood vessel membranes. After using small interfering RNA to knockdown NOX4, the proliferation of PASMCs was inhibited and ROS level was decreased. This indicated that NOX4, as an important factor, is involved in pulmonary vascular remodeling during pulmonary hypertension development [27]. Talija Djordjevic, etc. confirmed that the proliferation of human PASMCs is associated with NADPH oxidase, and the role is mainly acted by NADPH oxidase NOX4 [28]. Isabel Diebold, etc. also found that in hypoxia-induced PASMCs proliferation, there are NOX4 up-regulation and increased ROS levels [29]. In addition, by using Mn-TBAP to clear whether ROS can reverse hypoxia -induced PASMCs proliferation [30], the hydrogen peroxide can promote cell proliferation by regulating calcium homeostasis in PASMCs [31]. Our results consistently showed that BMP4 up-regaluted ROS level and proliferation of rat PASMCs, and NOX4 knockdown significantly reduced BMP4 increased ROS production and cell proliferation. These results suggest that NOX4 has a proportional relationship with ROS levels, and ROS plays a key role in regulating the proliferation and differentiation of PASMCs.

However, in rat distal PASMCs, it remains unclear through which signaling pathways do NOX4 and ROS regulate intracellular calcium homeostasis, thereby promote cell proliferation. Ziying Wang, etc. showed that the NOX4 -derived ROS up-regulates TRPC6 expression in puromyc in aminonucleoside-induced nephropathy, which subsequently regulates the intracellular calcium homeostasis [32]. A study about podocytes found that insulin promotes ROS generation through NOX4, and thus facilitates TRPC6 expression [33]. Our previous studies have revealed that TRPC1 and 6 could be increased by BMP4 treatment and associated with the SOCE regulation and the proliferation in PASMCs. Our present study also found that silencing NOX4 gene could down-regulate TRPC1 and 6 expression, basal [Ca^2+^]_i_ and SOCE in PASMCs, additionally, recombinant BMP4 treatment still cannot recover or up-regulated TRPCs expression, basal [Ca^2+^]_i_ and SOCE. This indicates that the regulation of BMP4 on TRPC1 and 6 expressions may be mediated by promoting NOX4-induced ROS. External ROS recovered the effects of NOX4 knockdown. It is possible that BMP4 acts as NOX4 upstream and regulates intracellular calcium through NOX4.

In conclusion, this article originally demonstrated that in rat distal PASMCs, NOX4 acts as a mediator to facilitate BMP4 increased TRPC1 and 6 expression and the regulation of intracellular calcium through TRPC channels, thereby promoting cell proliferation and vascular remodeling.
